# Assessment of the Vanillin Anti-Inflammatory and Regenerative Potentials in Inflamed Primary Human Gingival Fibroblast

**DOI:** 10.1155/2021/5562340

**Published:** 2021-05-04

**Authors:** Erica Costantini, Bruna Sinjari, Katia Falasca, Marcella Reale, Sergio Caputi, Srinivas Jagarlapodii, Giovanna Murmura

**Affiliations:** ^1^Department of Medical, Oral and Biotechnological Science, University G.d'Annunzio, Chieti-Pescara, Italy; ^2^Department of Innovative Technologies in Medicine and Dentistry, University G.d'Annunzio, Chieti-Pescara, Italy; ^3^Department of Medicine and Aging Science, University G.d'Annunzio, Chieti-Pescara, Italy

## Abstract

**Background:**

Inflammatory responses have been associated with delayed oral mucosal wound healing and the pathogenesis of the periodontal disease. The invasion of microbes into the tissues and the establishment of a chronic infection may be due to impaired healing. The protracted inflammatory phase may delay wound healing and probably support tissue fibrosis and reduce tissue regeneration. Vanillin is a well-known natural compound with potential anti-inflammatory capacity. Hence, we hypothesized that Vanillin could accelerate wound healing reducing inflammation and especially cytokine production making the oral tissue repair process easier.

**Methods:**

Our hypothesis was tested using primary human gingival fibroblast (HGF) cell pretreated with Vanillin and primed with IL-1*β*, as inductor of proinflammatory environment. After 24 hours of treatments, the gene expression and production of IL-6, TNF-*α*, IL-8, COX-2, iNOS, and nitric oxide (NO) generation and the wound healing rate were determined.

**Results:**

In IL-1*β*-primed cells, preincubation with Vanillin reduced IL-6, IL-8, COX-2, and iNOS expression and NO release, compared to IL-1*β*-primed cells. Moreover, Vanillin determines the increased gene expression of nAChR*α*7, leading us to hypothesize a role of Vanillin in the activation of the cholinergic anti-inflammatory pathway. Furthermore, in presence of mechanical injury, the Vanillin preincubation, wound closure may be reducing the expression and release of IL-6 and TNF-*α* and upregulation of COX-2 and IL-8.

**Conclusion:**

Together, the results of this study highlight the anti-inflammatory and tissue repair ability of Vanillin in IL-1*β*-primed HGF. Therefore, Vanillin shows a potential therapeutic interest as an inflammatory modulator molecule with novel application in periodontal regeneration and oral health.

## 1. Introduction

Inflammation is defined as an essential biological event occurring for the defence of the body. It involves immune cells and multiple mechanisms that operate at different levels, including alterations in immune cell types in tissues, changes in cellular reactivity to inflammatory stimuli, regulation of signaling pathways, and control of gene expression [1]. In fact, macrophages promote the innate host defence and inflammatory reaction, with the release of inflammatory mediators like interleukin- (IL-) 1*β*, IL-6, IL-8, tumour necrosis factor- (TNF-) *α*, reactive oxygen species (ROS), and nitric oxide (NO) [2]. These molecules mediate the inflammatory response and trigger adaptive immune activation, through the interaction with cellular specific receptors [3]. Periodontal diseases, inflammatory conditions with an infectious aetiology including gingivitis and periodontitis, are amongst the prevalent oral health illnesses that may ultimately lead to severe chronic conditions in the oral cavity [4]. Inflammation seems to be linked to microorganism growth associated with the destruction of oral tissues and release of harmful nutrients, such as degraded collagen, heme-containing compounds, sources of amino acids, and iron. These events can drive the establishment of a proinflammatory microenvironment and the production of oxidative stress mediators with the periodontal pocket formation and gingival tissue, alveolar bone, and periodontal ligament destruction. Therefore, the prominent role of the inflammatory response in periodontal disease pathogenesis suggests that reduction of bacterial load and regulation of inflammation are the main goals for the treatment of periodontal disease. Recently, it has become clear that natural compounds are important regulators of immune responses [5]. To date, natural phenolic compounds have gained considerable attention for the improvement of human health. The increase in the use of herbal medicines has renewed interest in the effects of plant extracts for the control of plaque and other oral diseases [5–7]. In their review, Kouidhi et al. [8] documented the potential use of plant extracts, essential oils, and natural compounds as biofilm preventive agents in dentistry, and the search for natural anti-inflammatory agents with fewer side effects has made the leap from research laboratories to the pharmaceutical industry. Different natural common herb components such as the tea tree oil, aloe vera, vanillin, curcumin, and chamomile have been recently introduced as anti-inflammatory molecules for *dental* treatments [9].

Vanillin (4-hydroxy-3-methoxybenzaldehyde) is the major component of natural vanilla, which is one of the most widely used flavor components in food and personal products [10] with antimicrobial, antimutagenic, and antiangiogenic effects [11, 12]. Many studies have investigated the role of Vanillin in nervous systems, demonstrating the protection against rotenone-induced neurotoxicity in SH-SY5Y cells [13] or the antineuroinflammatory properties in microglial cells [14]. Keeping in mind the antimicrobial and anti-inflammatory capacity of Vanillin and since to date, there are no reports on the effects of Vanillin on the oral tissue cells, this study is aimed at investigating the ability of Vanillin to modulate the inflammatory response, oxidative stress, and oral tissue repair. Human gingival fibroblasts (HGFs) are the most abundant resident cells in the oral mucosa, and inflammatory cytokine produced by HGF may have a central role during gingival inflammation and periodontal tissue repair. Thus, to mimic in vitro the inflammatory microenvironment in periodontal disease, we used IL-1*β* as inductor and enhancer of the proinflammatory response on primary gingival fibroblasts [15]. With this in vitro model, we aim to evaluate the effects of Vanillin on inflammation, oxidative stress, and tissue repair, in order to open a way for its potential use in periodontal diseases.

## 2. Materials and Methods

### 2.1. HGF Sampling

Healthy patients, aged between 20 and 25 years, were recruited at the Dental Clinic of “G.d'Annunzio” of Chieti-Pescara University, Chieti, Italy, for wisdom tooth extraction. After written informed consent release, discarded gingival tissues were obtained and processed for primary human gingival fibroblast (HGF) isolation. The gingival tissues were placed in physiological solution, at room temperature, for transport to the cell culture laboratory. The study was approved with the committee report no. 14, on 23 July 2015 by the Inter-Institutional Ethic Committee of the University of Chieti-Pescara, Italy. Each sample was coded to guarantee the anonymity of the donors.

### 2.2. Cell Isolation and Culture

For each healthy donor, gingival fragments were washed with physiological sodium chloride solution and placed in a T25 culture flask (Merck KGaA, Darmstadt, Germany) filled with Dulbecco's Modified Eagle's Medium (DMEM) (pH 7.2; Merck KGaA, Darmstadt, Germany) supplemented with 10% heat-inactivated fetal bovine serum (FBS), 100 U/mL penicillin, 100 *μ*g/mL streptomycin, and 2 mmol/L l-glutamine (Merck KGaA, Darmstadt, Germany) and left in a humidified CO_2_ incubator set at 37°C till cell adhesion occurred. The culture media were replaced with fresh medium twice a week for 15 days to obtain primary HGF. Cells were collected after adding of 1X trypsin-EDTA solution (Merck KGaA, Darmstadt, Germany) and used for subsequential experiments. Primary HGFs were used for experiments between passages 4 and 5 after isolation.

### 2.3. Cell Viability Assay

HGFs were seeded into the wells of the 96-well plates at a density of 0.4 × 10^3^ cells/well. The following day, IL-1*β* (Peprotech, Rocky Hill, USA) within a concentration range 0.1–10 ng/mL or Vanillin (Merck KGaA, Darmstadt, Germany) within a concentration range 100-300 *μ*M or negative control (0.1% (v/v) DMSO) and positive control (100% (v/v) DMSO) were added to the attached cells, in presence of fresh culture medium, for 24 h. Cell viability was determined by the 3-(4,5-dimethylthiazol-2-yl)-2,5-diphenyltetrazolium bromide (MTT) assay, according to the recommendations of the manufacturer (Merck KGaA, Darmstadt, Germany). At the end of incubation, MTT reagent was added for 2 h and incubated at 37°C, the absorbance was measured at OD590 nm. Absorbance data were normalized to the untreated control group, considered as 100% of viability.

### 2.4. In Vitro Treatments

The inflammatory condition of periodontal disease was mimic *in vitro* priming HGFs with IL-1*β* (1 ng/ml). To evaluate the ability of Vanillin on inflammatory response modulation, we have designed two different experimental conditions. In the first experimental condition, 0.15 × 10^5^ cells/cm^2^ HGF were preincubated for 2 h with 200 *μ*M of Vanillin and treated with IL-1*β* for the following 24 h of incubation at 37°C, 5% CO_2_, and then, gene expression and production of inflammatory mediators were evaluated. In the second experimental condition, HGFs were seeded at a cell density of 0.15 × 10^5^ cells/cm^2^ in two sets of 6-well plates in a complete medium (10% FBS) and grown until the monolayer was confluent. Subsequently, to mimic the oral wound, a scratch was made mechanically with a 10 *μ*l sterile pipette tip. Detached cells and debris were removed by washing with Dulbecco's Phosphate Buffered Saline (PBS), fresh medium containing IL-1*β*, or Vanillin was added to carry on the incubation at 37°C, 5% CO_2_ for 24 h. Alternatively, Vanillin was added 2 h before the scratch, and the added fresh medium was supplemented with Vanillin+IL-1*β*. The effect of Vanillin on the rate of scratched monolayer closure was monitored by observing the cell repopulation of the area between the wound edges, using an inverted phase-contrast microscope (Leica, Germany) equipped with a CCD camera, and the remaining cell-free area was measured.

At the end of incubation, the cells were harvested for analysis of inflammatory mediators involved in wound healing.

### 2.5. Nitrite Determination

To measure the NO production, nitrite concentration in the culture supernatant was determined using Griess reagent (1% sulfanilamide and 0.1% N-(1-naphthyl)-ethylenediamine dihydrochloride in 5% H3PO4) (Cayman Chemical, Ann Arbor, Michigan, USA). 100 *μ*l of cell culture supernatant and 100 *μ*l Griess reagent were mixed and incubated for 10 min, to color development. The absorption was estimated at 540 nm, using a Glomax Multireader spectrophotometer (Promega, Madison, WI, USA). Nitrite standard (Cayman Chemical, Ann Arbor, Michigan, USA) was used to generate a standard curve for quantification. Results were obtained from three independent experiment measurements.

### 2.6. ELISA Analysis

The culture supernatants were quantitatively assayed for IL-6, TNF-*α*, and IL-8 (BOOSTER PicokineTM ELISA, Boster Biological Technology, Pleasanton, CA, USA), with concentrations following the manufacturer's instructions. Optical density was measured at 450 nm. The inflammatory cytokine levels were determined in all the different condition samples through duplicated measurements requiring 100 *μ*L of culture supernatant. Results were standardized by using internal controls supplied with each kit, with a known concentration of the target protein. For IL-6, sensitivity < 0.3 pg/ml; for TNF-*α* and IL-8, sensitivity < 1 pg/ml.

### 2.7. RNA Isolation and Real-Time RT-PCR Analysis

Total RNA was isolated using the classic phenol-chloroform method. Total RNA was quantified at 260 nm using NanoDrop 2000 ultraviolet-visible (UV-Vis) spectrophotometer (Thermo Fisher Scientific, Waltham, MA, USA). 1 *μ*g of RNA was reverse transcribed to cDNA for 15 min at 42°C and 3 min at 90°C to inactivate Quantiscript Reverse Transcriptase, according to the protocol of QuantiTectReverse Transcription Kit (Qiagen, Hilden, DE). Real-time PCR was performed in a CFX Real-Time PCR Detection Systems (Bio-Rad, Hercules, California, USA), using GoTaq® qPCR Master Mix (Promega, Madison, WI, USA) to evaluate the gene expression of IL-6, nAChR*α*7, TNF-*α*, IL-8, iNOS, COX-2, and 18S housekeeping gene (Table 1). The amplification program consisted of a preincubation step for cDNA denaturation (2 min 95°C), followed by 40 cycles consisting of a denaturation step (30 s 95°C), an annealing step (60 s 60°C), and an extension step (30 s 68°C). At the end of each run, melting curve was performed in the temperature range of 60 to 95°C. Expression levels for each gene were performed according to the 2^-*ΔΔ*Ct^ method.

### 2.8. Statistical Analysis

Quantitative variables are summarized as the mean value and standard deviations (SD) in the figures. Precision of the fold change, calculated with 2^–*ΔΔ*Ct^ method, was determined using the 95% confidence interval (95% CI). Student *t*-test for unpaired sample was applied to evaluate statistical differences. Tests threshold of will be assumed equal to *p* value ≤0.05. Analyses were performed by the SPSS Inc. statistical software package (Version 23.0). One, two, and three symbols represent a significant difference between two groups with *p* ≤ 0.05, *p* < 0.01, and *p* < 0.001, respectively.

## 3. Results

### 3.1. Effects of Vanillin and IL-1*β* on HGF Viability

**Figure 1:**
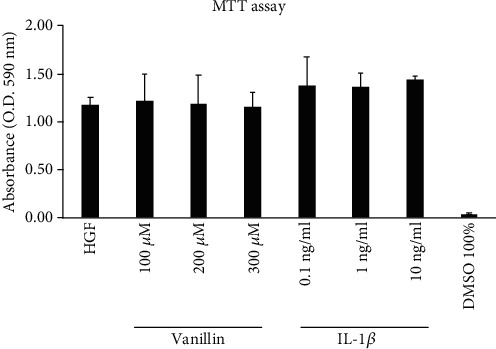
MTT viability assay in HGF cell treated for 24 h with Vanillin (100, 200, and 300 *μ*M) or IL-1*β* (0.1, 1, and 10 ng/ml). Absorbance values are given as media ±SD of three independent experiments.

Preliminarily, using 3-(4,5-dimethylthiazol-2-yl)-2,5-diphenyltetrazolium bromide (MTT) assay, we analyzed the effects of Vanillin (100, 200, and 300 *μ*M) and IL-1*β* (0.1, 1, and 10 ng/ml), on HGF survival. The dose-response experiments suggested that cell viability after 24 h of incubation with Vanillin or IL-1*β* was not affected at any of the tested concentrations (Figure 1). Thus, the concentration of 1 ng/mL of IL-1*β* and 200 *μ*M of Vanillin were selected for all the following experiments.

### 3.2. Production of Inflammatory Mediators

**Figure 2:**
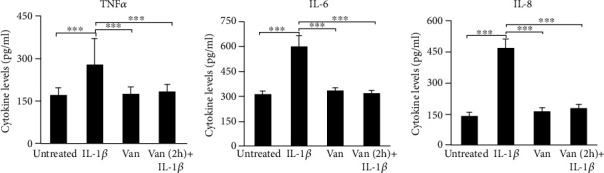
TNF-*α*, IL-6, and IL-8 levels in HGF cell culture supernatant. The mean values ± SD were reported, ^∗∗∗^*p* < 0.001. All experiments were repeated in triplicate.

To investigate the effects of Vanillin, on inflammation, we have used IL-1*β* to promote the proinflammatory response in HGF cells. After 24 h incubation of HGF with IL-1*β*, Vanillin alone or Vanillin (2 h pretreatment) plus IL-1*β*, IL-6, IL-8, and TNF-*α* levels were evaluated in cell culture supernatant, by ELISA assay. TNF-*α*, IL-6 (*p* < 0.001), and IL-8 (*p* < 0.001) were upregulated by the treatment with IL-1*β*. Meanwhile, in presence of Vanillin, alone or as pretreatment of inflamed HGF, a significant reduction of TNF-*α*, IL-6, and IL-8 was observed compared to IL-1*β* primed cells (*p* < 0.001) (Figure 2).

### 3.3. Gene Expression of Inflammatory Mediators

**Figure 3:**
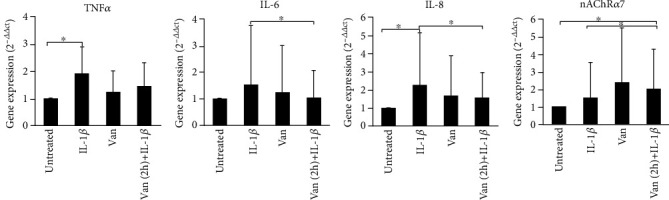
Gene expression of TNF-*α*, IL-6, IL-8, and nAChR*α*7 in HGF cells. Data are reported as mean and 95% CI, of three independent experiments. ^∗^*p* < 0.05. IL-1*β*: IL-1*β*-primed cells; Van: Vanillin; Van (2 h)+IL-1*β*: Vanillin (2 h pretreatment) plus IL-1*β*.

In order to investigate if differences in supernatant levels of cytokine mirror a different gene expression profile of proinflammatory cytokine, we have evaluated the cytokine expression in treated and untreated HGF, using real-time PCR. TNF-*α* gene expression levels were significantly (*p* = 0.047) induced in IL-1*β*-primed HGF, such as levels of IL-8 (*p* = 0.037) compared to the untreated cells. Treatment with Vanillin does not significantly affect gene expression levels, compared to the other conditions. Unlike the pretreatment with Vanillin, for 2 h, reduces the expression of IL-6 (*p* = 0.049) and IL-8 (*p* = 0.042) with respect to IL-1*β*-primed HGF. Moreover, due to the nAChR*α*7 can represent an important marker for the stabilization of tissue homeostasis in the presence of persistent chronic inflammation, we have evaluated Vanillin effects on nAChR*α*7 gene expression. As shown in Figure 3, IL-1*β*-primed HGF showed no statistically significant variation in nAChR*α*7 expression levels (*p* > 0.05) with respect to untreated cells. Meanwhile, the Vanillin alone led to a slight increase of nAChR*α*7 gene expression, and in Vanillin-pretreated HGF cells, the nAChR*α*7 mRNA expression was significantly increased, compared to untreated cells (*p* = 0.049) and to the IL-1*β*-primed HGF (*p* = 0.42).

### 3.4. COX-2 and iNOS Gene Expression and NO Production

**Figure 4:**
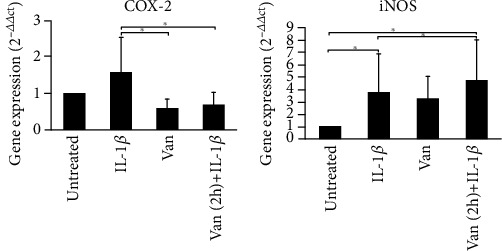
Gene expression of COX-2 and iNOS in HGF cells. Data are reported as mean and 95% CI, of three independent experiments, ^∗^*p* < 0.05. IL-1*β*: IL-1*β*-primed cells; Van: Vanillin; Van (2 h)+IL-1*β*: Vanillin (2 h pre-treatment) plus IL-1*β*.

Inflammatory stimuli induce the COX-2 and iNOS expression in the sites of inflammation and damaged tissue; thus, we have evaluated the effect of Vanillin on IL-1*β*-primed HGF. Both HGFs treated with Vanillin alone or with Vanillin (2 h pretreatment) plus IL-1*β* showed a significant downregulation of COX-2 gene expression (*p* = 0.42) with respect to IL-1*β*-primed HGF. The iNOS gene expression, responsible also for NO production, was significantly upregulated in all the treatment conditions, with higher increase in Vanillin pretreatment of inflamed HGF (Figure 4).

**Figure 5:**
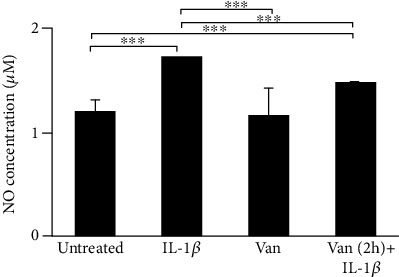
Levels of NO in HGF cells were determined based on Griess reagent reaction. Experiments were conducted in duplicate, and changes were reported as mean ± SD; ^∗∗∗^*p* < 0.001. IL-1*β*: IL-1*β*-primed cells; Van: Vanillin; Van (2 h)+IL-1*β*: Vanillin (2 h pretreatment) plus IL-1*β*.

In addition, as shown in Figure 5, the production of NO was significantly increased in HGF IL-1*β* primed, compared to untreated cells. Vanillin pretreatment of IL-1*β*-primed HGF cells downregulates NO production levels, compared to IL-1*β*-primed HGF cells. These results underline the ability of Vanillin to regulate NO production acting by iNOS inhibition. In addition, as shown in Figure 5, the production of NO was significantly increased in HGF IL-1*β* primed, compared to untreated cells. Vanillin pretreatment, in IL-1*β*-primed HGF cells, showed a downregulation of NO production levels, compared to IL-1*β*-primed HGF cells. These results underline the ability of Vanillin to regulate NO production acting by iNOS inhibition.

### 3.5. Effect of Vanillin Pretreatment on Inflamed HGF Scratched Cells

#### 3.5.1. Wound Healing

**Figure 6:**
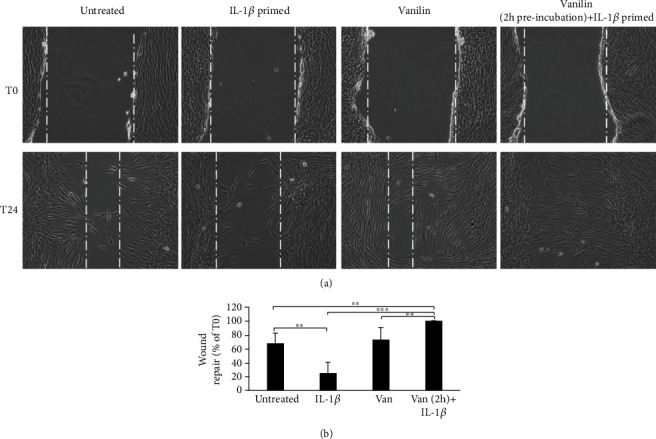
Wound healing of scratched oral fibroblast monolayer photographs and measurement of wound area were made immediately after the scratch (T0) and after 24 h (T24). (a) Representative images of wound-healing assay. (b) Wound repair was evaluated measuring the remaining cell-free area after 24 h and expressed as a percentage of the initial wound size (T0) assumed as 100%. ^∗∗^*p* < 0.01; ^∗∗∗^*p* < 0.001. IL-1*β*: IL-1*β*-primed cells; Van: Vanillin; Van (2 h)+IL-1*β*: Vanillin (2 h pretreatment) plus IL-1*β*.

Infections are the primary factors underlying inflammation, but also, injury or trauma can trigger inflammatory responses. The wound-healing assay was used to study the molecular mechanisms of tissue repair, as well as to study potential application of Vanillin as treatment to improve soft tissue healing. As showed in Figure 6(a), an increased HGF migration was observed in Vanillin-pretreated cells after 24 h scratching, reaching the 100% of wound closure. In IL-1*β*-primed cells, an impaired coverage of cell-free area (25% reduction of wound size compared to T0) was detected (Figures 6(a) and 6(b).

#### 3.5.2. Inflammatory Mediator Expression and Production during Wound Healing

**Figure 7:**
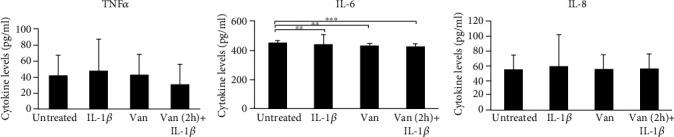
Levels of TNF-*α*, IL-6, and IL-8 in supernatant of HGF scratched cells. Data are reported as mean and 95% CI, of three independent experiments. ^∗∗^*p* < 0.01; ^∗∗∗^*p* < 0.001. IL-1*β*: IL-1*β*-primed cells; Van: Vanillin; Van (2 h)+IL-1*β*: Vanillin (2 h pretreatment) plus IL-1*β*.

The repair process is mediated by the interaction of molecular signals, which orchestrate the cellular activities underling inflammation and healing. Thus, we have determined the effect of Vanillin on production and gene expression of proinflammatory mediators. The preincubation with Vanillin in the scratched HGF monolayer reduced the production of IL-6 compared to IL-1*β*-primed cells (*p* = 0.037) and with respect to the untreated cells (*p* = 0.004), whereas the production of TNF-*α* and IL-8 was not significantly modified (Figure 7).

**Figure 8:**
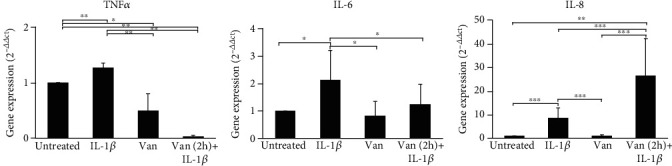
Gene expression of TNF-*α*, IL-6, and IL-8 in HGF scratched cells. Data are reported as mean and 95% CI, of three independent experiments. ^∗^*p* < 0.05; ^∗∗^*p* < 0.01; ^∗∗∗^*p* < 0.001. IL-1*β*: IL-1*β*-primed cells; Van: Vanillin; Van (2 h)+IL-1*β*: Vanillin (2 h pretreatment) plus IL-1*β*.

On the other hand, the gene expression of TNF-*α* and IL-8 was significantly affected by the treatments. In particular, a reduction of TNF-*α* in HGF scratched cells was induced by Vanillin (*p* = 0.010) and even more with Vanillin (2 h pretreatment) plus 1*β* cells (*p* = 0.002) (Figure 8). The IL-6 gene expression was reduced in cells treated with Vanillin (*p* = 0.043) and downregulated by Vanillin (2 h pretreatment) plus 1*β* (*p* = 0.049), compared to 1*β*-primed cells. In the scratched HGF monolayer, we observed higher levels of IL-8 gene expression in Vanillin (2 h pretreatment) plus 1*β*, with respect to both untreated (*p* < 0.001) and IL-1*β*-primed cells (*p* < 0.001).

#### 3.5.3. Vanillin Effect on iNOS and COX-2 Gene Expression and NO Production in Inflamed HGF Scratched Cells

**Figure 9:**
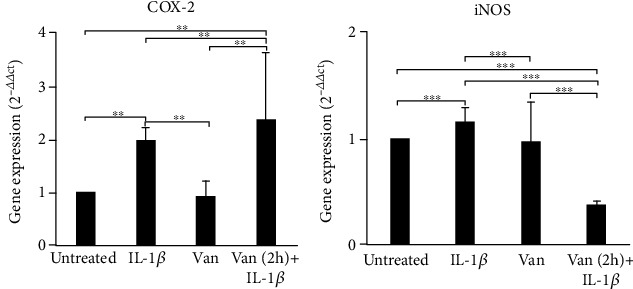
Gene expression of COX-2 and iNOS in HGF cells. Data are reported as mean and 95% CI, of three independent experiments. ^∗∗^*p* < 0.01; ^∗∗∗^*p* < 0.001. IL-1*β*: IL-1*β*-primed cells; Van: Vanillin; Van (2 h)+IL-1*β*: Vanillin (2 h pretreatment) plus IL-1*β*.

A cross-talk between NOS and COX enzymes, key inflammatory mediators, has been suggested [16]. Thus, we have evaluated the mRNA expression levels of COX-2 and iNOS in scratched HGF cell monolayer preincubated with Vanillin and primed with IL-1*β*. After 24 h of incubation in presence of mechanical damage and treatments, a significant increase of COX-2 gene expression (*p* < 0.01) was detected in Vanillin (2 h pretreatment) plus 1*β*, compared to other conditions (Figure 9). Meanwhile, Vanillin (*p* = 0.022) alone or as pretreatment (*p* = 0.024) of inflamed HGF showed a significant upregulation of iNOS, with respect to 1*β*-primed cells.

**Figure 10:**
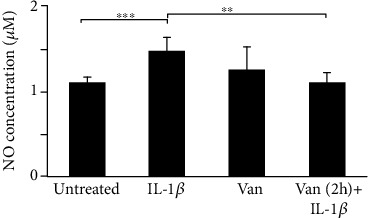
Levels of NO in HGF cells were determined based on Griess reagent reaction. Experiments were conducted in duplicate, and changes were reported as mean ± SD. ^∗∗^*p* < 0.01; ^∗∗∗^*p* < 0.001. IL-1*β*: IL-1*β*-primed cells; Van: Vanillin; Van (2 h)+IL-1*β*: Vanillin (2 h pretreatment) plus IL-1*β*.

The weak induction of NO production observed in inflamed HGF scratched cells (*p* < 0.001) was reverted by Vanillin (2 h pretreatment) plus 1*β* (*p* = 0.028). Reduction is *p* = 0.028, as shown in Figure 10.

## 4. Discussion

The interest in the therapeutic potential of phenolic compounds, present in food and medicinal plants, had an increased relevance in the last decade [15, 17]. In many diseases, local inflammation could determine systemic inflammation, characterized by systemic oxidative stress, activation of circulating inflammatory cells, and increased circulating levels of inflammatory cytokines. The influence of inflammatory response by phenolic compounds has become the focus of several new treatment strategies with promising results [18, 19]. The use of Vanillin in oral inflammatory diseases has not yet been evaluated, although its interesting effects on oxidative stress and inflammation may represent a new strategy for the treatment of inflammatory diseases. Oral fibroblasts, other than by macrophages, dendritic cells, epithelial, and keratinocytes cells, have a central role in the inhibition of bacterial products and proinflammatory cytokine production [20–22]. In periodontitis, proinflammatory cytokines, such as IL-1*β*, IL-6, IL-8, and TNF-*α*, seem to be the major mediators, involved in the destruction of periodontal tissue. Individuals with periodontal infections show high concentrations of circulating inflammatory markers that directly correlated with the severity of tissue destruction and inflammatory serum markers [20–23]. High levels of IL-6 and TNF-*α*, important mediators in the switch from acute to chronic inflammation, are present in periodontal gingival crevicular fluid and gingival tissues, and after nonsurgical periodontal therapy, reduced levels of TNF-*α*, IL-1*β*, and IL-6 were observed, confirming their role in periodontal disease [24, 25]. In this study, we have simulated the proinflammatory microenvironment by IL-1*β* priming of HGF and we have evaluated the effect of Vanillin on inflammatory mediator gene expression and production. Our results showed that in IL-1*β*-primed HGF cells, Vanillin pretreatment decreases expression and production of proinflammatory mediators, such as IL-8, IL-6, and TNF-*α*. Progression of periodontal disease may be derived by NO and COX-2 signaling pathways [26, 27]. In Vanillin pretreated cells, we detected a reduction of iNOS and COX-2 gene expression and NO production. Together, these results underline the ability of Vanillin to modulate inflammatory response in HGF-inflamed cells. nACh*α*7 receptor is a critical regulator of the “cholinergic anti-inflammatory pathway” [28, 29] and is expressed in HGF cells. Thus, we investigate if the anti-inflammatory ability of Vanillin may be linked to nAChR*α*7 expression. Our results showed the Vanillin-induced increase of nAChR*α*7 expression in accord with the reduction of proinflammatory cytokine gene expression. Taking into account that inflammatory mediators drive the onset and progression of connective tissue degradation, our second goal was to analyze the effect of Vanillin on wound healing [30–32]. Vanillin, in our in vitro model of wound healing, quickens wound closure. Moreover, the gene expression of IL-1*β*-induced mediators (IL-6 and TNF-*α*) in scratched HGF monolayer was reduced in Vanillin-pretreated cells, while no significant difference on IL-6, TNF-*α*, and IL-8 production was observed. Interestingly, in mechanical damaged IL-1 *β*-priming HGF, IL-8 gene expression was increased in presence of Vanillin pretreatment, as a response to a double mechanical and inflammatory stimulation. In fact, in vivo and in vitro studies have been reported that IL-8 induces neutrophil recruitment in the damaged area, stops the bacterial invasion, and promotes local neoangiogenic, cell proliferation, and tissue reepithelization in the hell of human wounds [33, 34].

IL-1*β* is known to increase COX-2 expression and prostaglandin (PG) production in HGF [35–37]. In the present study, we observed that the IL-1*β* upregulation of COX-2 expression was increased by Vanillin (pretreatment) plus 1*β* treatment, in accord with the role of COX-2 in early and late regulation of the outcome of wound repair [35–37]. iNOS expression occurs very early after tissue injury, suggesting that this enzyme is necessary in the early inflammatory phase; in fact, our results showed a reduction of iNOS and NO at 24 h, when the complete wound healing was detected.

## 5. Conclusions

Overall, our results suggest that Vanillin may be useful in the management of the inflammatory periodontal disease by decreasing the excessive production of proinflammatory mediators and free radicals, as well as stimulating tissue regeneration and the management of soft tissue restoration. It is known that anti-inflammatory and antioxidant molecules added to oral hygiene products improve the indexes of periodontal disease [38, 39], with a reduction of the risk of associated systemic diseases. Thus, Vanillin could be potentially used in formulations suitable for oral application as a nonpharmacological agent for periodontal health and also as integral care of several disease managements, such as people with diabetes or HIV-infection, with tissue healing delayed rate.

## Figures and Tables

**Table 1:** Primer sequences used for real-time PCR reactions.

Gene	Forward primer sequence (5′-3′)	Reverse primer sequence (5′-3′)	Amplicon length
18s	CTTTGCCATCACTGCCATTAAG	TCCATCCTTTACATCCTTCTGTC	199 bp
IL-6	GTACATCCTCGACGGCATC	ACCTCAAACTCCAAAAGACCAG	198 bp
TNF-*α*	CCTTCCTGATCGTGGCAG	GCTTGAGGGTTTGCTACAAC	184 bp
IL-8	GTGTAAACATGACTTCCAAGCTG	GTCCACTCTCAATCACTCTCAG	182 bp
iNOS	GGTATCCTGGAGCGAGTGGT	CTCTCAGGCTCTTCTGTGGC	212 bp
COX-2	GACAGTCCACCAACTTACAATG	GGCAATCATCAGGCACAGG	105 bp
nAChR*α*7	CTGCTCGTGGCTGAGATCAT	CTGGTCCACTTGGGCATCTT	167 bp

## Data Availability

The authors confirm that the data supporting the results of this study are available within the article and can be requested from the correspondent in case of further clarification.
